# Supplementary material for: Identifying and characterizing extrapolation in multivariate response data
Source: PLoS One. 2019 Dec 5;14(12):e0225715. doi: 10.1371/journal.pone.0225715 (PMC6894872; doi:10.1371/journal.pone.0225715)
Supplement: S1 Fig — (PDF) [file pone.0225715.s001.pdf]

S1 Figures: Violin plots of covariate densities and extrapolation points plotted.

In this paper, we present new methods for identifying extrapolation within a multivariate setting. In addition to visual exploration of extrapolation locations identified using various cutoff values, we have also explored the covariate space though a CART model to investigate whether certain covariates are more influential than others when determining whether a prediction is an extrapolation. In Fig 1 and Fig 2 below we have visualized a selection of the lakes identified as extrapolations. The (scaled) covariate values of the extrapolated lake locations are within the distribution of the data, with only a few exceptions (e.g. IWS_lk_ratio, a measure of the watershed to lake ratio). Rather than a few key variables standing out, it appears to be some combination of variables that makes a lake an extrapolation. Tables of unscaled covariate values for extrapolation lakes featured in the below figures are presented in S2 Tables.

Fig 1. Violin plots of densities for each covariate included in model. Covariate values have been scaled to have mean 0 and SD of 1. Data points represent lakes identified as extrapolations using MVPV(~~t~~D) and a 9~~5~~9% cutoff. The y-axis has been re-scaled to focus on the respective densities rather than long tails of several variables.

Fig 2. Violin plots of densities for each covariate included in model. Covariate values have been scaled to have mean 0 and SD of 1. Data points sets of matching colors each represent lakes identified as extrapolations using CMVPV~~(t)~~ for TN and a 9~~5~~9% cutoff. To increase readability of this plot a random sub-selection of 10 extrapolation lakes are plotted. The y-axis has been re-scaled to focus on the respective densities rather than long tails of several variables.
